# Congenital adrenal hyperplasia with associated giant adrenal myelolipoma, testicular adrenal rest tumors and primary pigmented nodular adrenocortical disease: A case report and brief review of the literature

**DOI:** 10.1016/j.radcr.2021.12.031

**Published:** 2021-12-28

**Authors:** Aaron Jacobson, Elaine Koberlein, Alan Thomay, Cara B Lombard, Ayodele Adelanwa, Dhairya A Lakhani, Kelly T. Smith

**Affiliations:** a Department of Radiology, West Virginia University, Morgantown, WV 26506, USA; b West Virginia School of Osteopathic Medicine, Lewisburg, WV, 24901; c Department of Surgical Oncology, West Virginia University, Morgantown, WV 26506, USA; d Department of Pathology, Anatomy and Laboratory Medicine, West Virginia University, Morgantown, WV 26506, USA

**Keywords:** Congenital adrenal hyperplasia, Adrenal myelolipoma, Testicular adrenal rest tumor, Primary pigmented nodular adrenocortical disease, CAH, congenital adrenal hyperplasia, TART, testicular adrenal rest tumor, PPNAD, primary pigmented nodular adrenocortical disease

## Abstract

Congenital adrenal hyperplasia is an autosomal recessive disease most commonly associated with 21-hydroxylase deficiency, an enzyme integral in the biosynthesis of mineralocorticoids and glucocorticoids. We present a case of a 49-year-old male with congenital adrenal hyperplasia and commonly associated findings of adrenal myelolipoma, testicular adrenal rest tumors, as well as primary pigmented nodular adrenocortical disease. Adrenal myelolipoma is a rare, benign disease process associated with exogenous steroid treatment noncompliance in the setting of congenital adrenal hyperplasia. Testicular adrenal rest tumors are benign testicular tumors associated with congenital adrenal hyperplasia. Primary pigmented nodular adrenocortical disease is an ACTH-independent cortisol producing lesion. Our case emphasizes the association of congenital adrenal hyperplasia with adrenal myelolipoma and testicular adrenal rest tumors as well as the importance of familiarity with these associations to guide patient management.

## Background

Primary pigmented nodular adrenocortical disease (PPNAD) is a rare benign adrenal condition characterized by ACTH-independent autonomous hypersecretion of cortisol, leading to Cushing syndrome, though manifestation is often subclinical [1]. The incidence of PPNAD is rare with only approximately 1%-2% of cases of Cushing's syndrome being caused by the disease entity [2,3]. The hypercortisolism from PPNAD is due to autonomous secretion from nodules arising in the adrenal zona reticularis. On gross appearance, the adrenal glands may be normal in size or enlarged and will contain multiple brown-black pigmented micronodules bilaterally, giving rise to the characteristic name of PPNAD. Nodules are unencapsulated with clear borders of demarcation due to adjacent atrophic cortex. Microscopically, cells are large and globular with clear or eosinophilic cytoplasm and may contain lipofuscin, which creates a granular brown pigment [4]. Cells will stain highly positive for synaptophysin, which suggests a neuroendocrine origin. PPNAD is associated with Carney Complex, a rare multiple endocrine neoplasm, which is characterized by pigmented cutaneous lesions, myxomatous tumors, and multiple endocrine tumors (including PPNAD) [3], [4], [5].

We report a case of PPNAD in a patient with history of classic congenital adrenal hyperplasia (CAH) and bilateral giant adrenal myelolipomas and testicular adrenal rests.

## Case report

We present a case of 49-year-old male with history of classic sub-type congenital adrenal hyperplasia, diagnosed at infancy, seen at the endocrinology clinic for salt craving, lightheadedness when working in hot conditions, increased pigmentation in sun exposed areas, and small testicular size. He reported normal facial hair growth and denied issues with libido. Reportedly, he was not on any medications for 20 years prior to presentation. His vitals were within normal limits. Pertinent laboratory workup was as follows: Androstenedione 2290 (normal 40-150 ng/dL) and 17-hydroxyprogesterone 8230 (normal <220 ng/dL). After this visit he was started on Prednisone (Deltasone) 2.5 mg, twice daily.

**Fig. 1:**
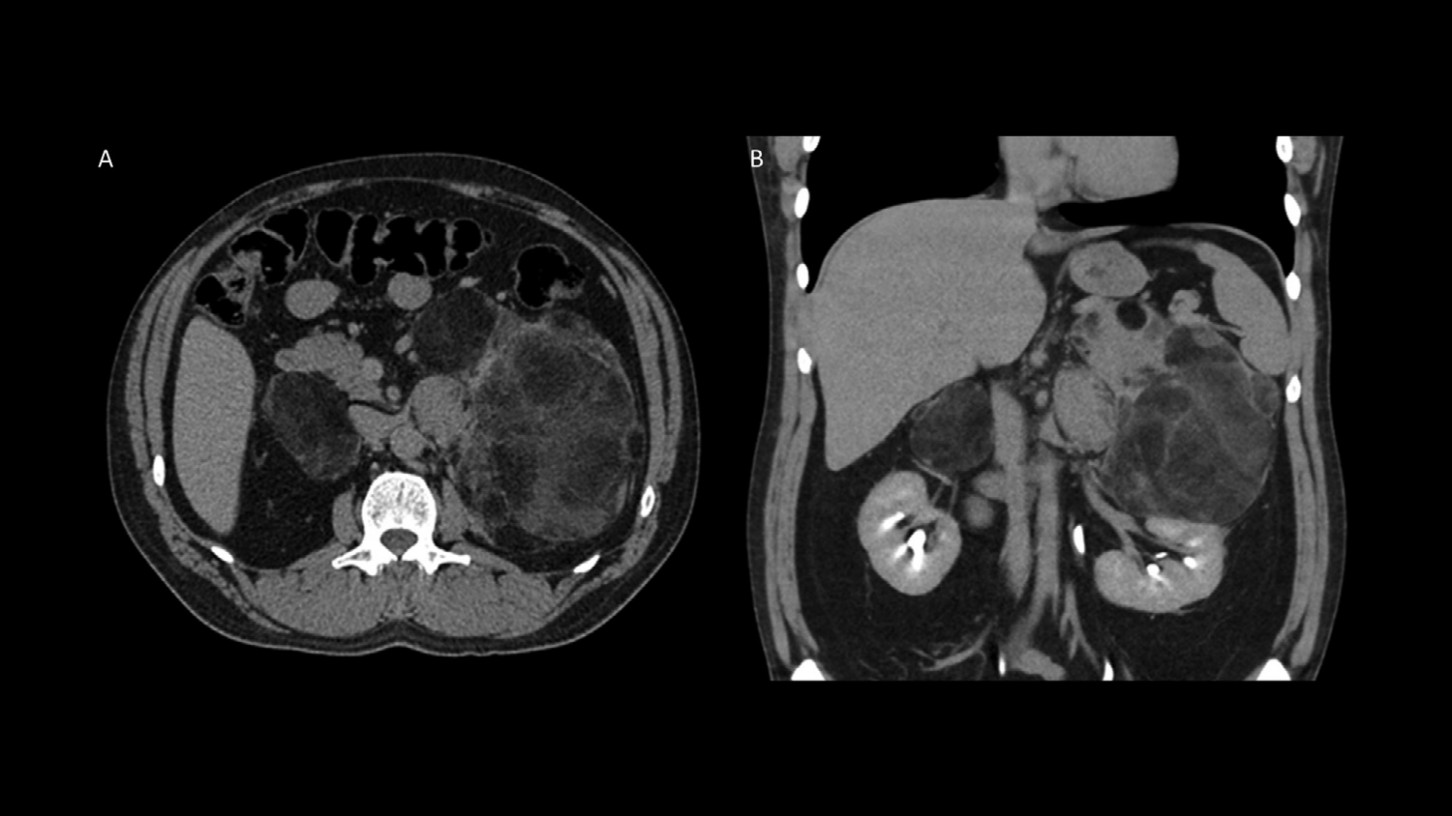
Axial (Fig. A) and Coronal (Fig. B) demonstrates multilobulated predominantly fatty tumor with interspersed weblike areas of soft tissue density. Findings were consistent with giant adrenal myelolipomas measuring 7.3 × 2.7 × 5.8 cm on the right and 18.0 × 13.4 × 12.0 cm on the left.

Further evaluation with CT of the abdomen and pelvis demonstrated multilobulated predominantly fatty masses with interspersed weblike areas of soft tissue density. Findings were consistent with giant adrenal myelolipomas measuring 7.3 × 2.7 × 5.8 cm on the right and 18.0 × 13.4 × 12.0 cm on the left (Fig. 1).

**Fig. 2:**
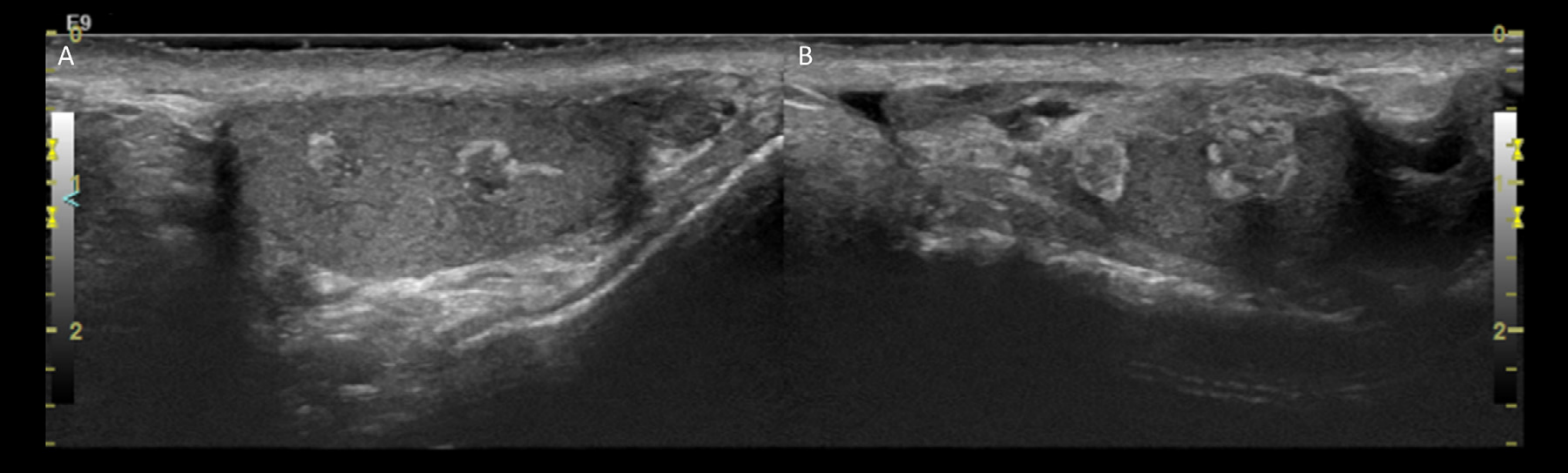
Transverse ultrasound of the bilateral testes reveals normal testicular size with numerous mixed echogenicity masses in the mediastinum testis ranging in size from 3 to 6 mm. Imaging features and clinical presentation were compatible with testicular adrenal rest tumors.

Ultrasound of scrotum was performed, which revealed numerous bilateral mixed hyperechoic and hypoechoic masses in the mediastinum testis. Imaging features and clinical presentation were compatible with testicular adrenal rest tumors (Fig. 2). The bilateral testes were normal in size with numerous bilateral mixed echogenicity masses in the mediastinum testis ranging in size from 3 to 6 mm. (Fig. 2)

**Fig. 3:**
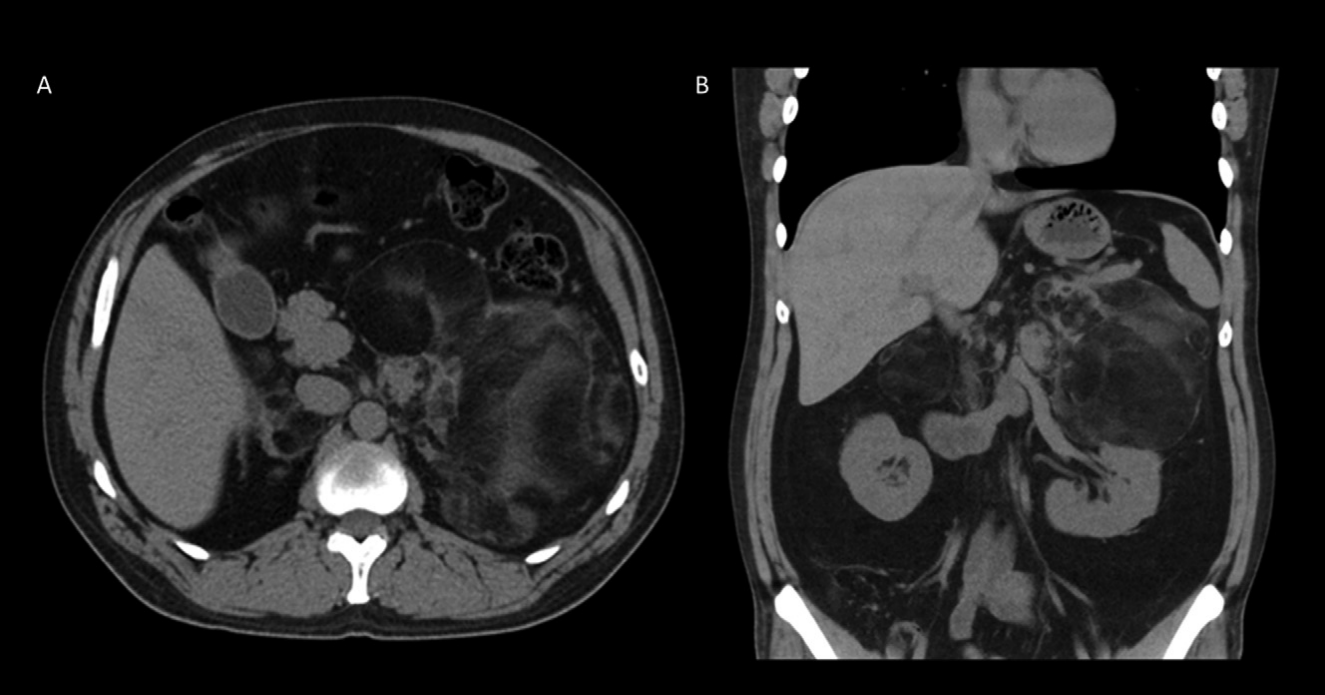
Follow up Axial (Fig. A) and coronal (Fig. B) demonstrates interval decrease in size of the giant left adrenal myelolipoma and overall unchanged size on the right with the right lesion measuring 7.1 × 4.2 × 5 cm and the left lesion measuring 16.8 × 12.0 × 10.4 cm.

**Fig. 4:**
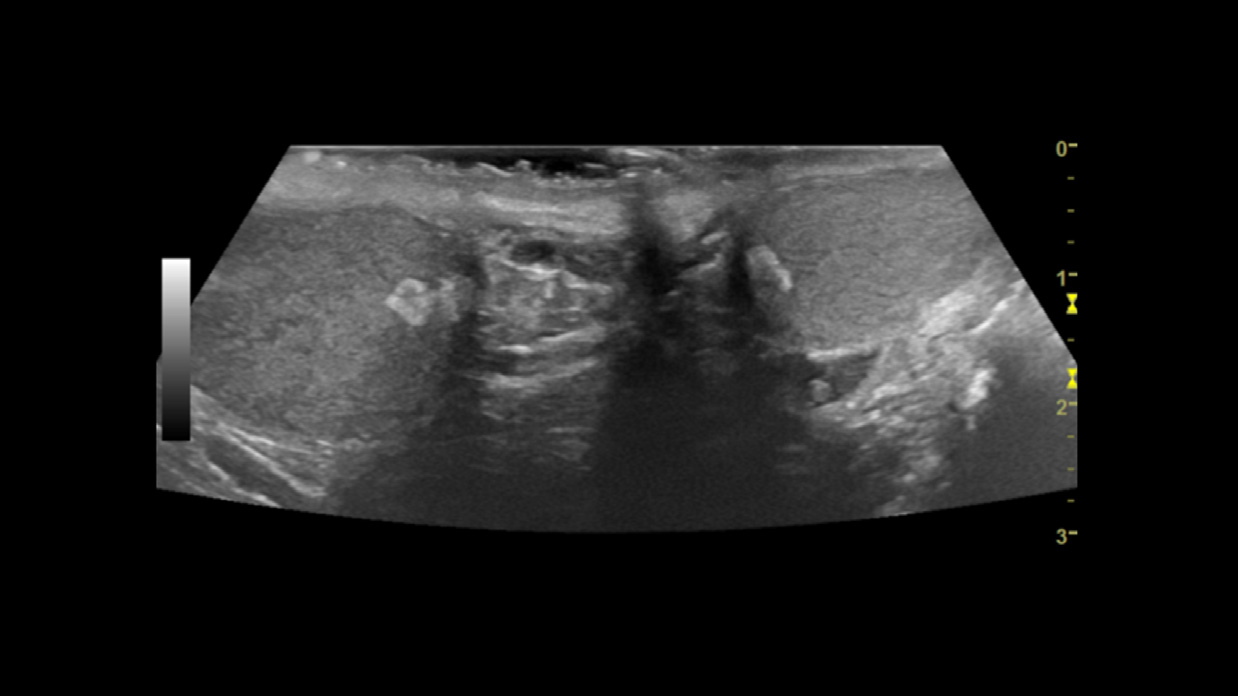
Follow up transverse testicular ultrasound in 2 mo displays unchanged size of the small mixed echogenicity masses in the bilateral testes.

Over the next several months, the patient was followed with interval laboratory values at one and two months. One month laboratory values of androstenedione of 870 (normal 40-150 ng/dL) and 17-hydroxyprogesterone 4170 (normal <220 ng/dL) and two-month laboratory values of Androstenedione of 73 (normal 40-150 ng/dL) and 17-hydroxyprogesterone of 289 (normal <220 ng/dL). With interval improvement in laboratory values, the patient was reimaged at two months with CT of the abdomen and pelvis, which demonstrated interval decrease in size of the large left adrenal myelolipoma and overall unchanged size on the right with the right mass measuring 7.1 × 4.2 × 5 cm and the left measuring 16.8 × 12.0 × 10.4 cm (Fig. 3). Remainder of the findings were stable. Ultrasound of the testes displayed unchanged size of the small mixed echogenicity masses in the bilateral testes (Fig. 4).

During the interim 3 months, the patient established with surgical oncology with chief complaint of back and abdominal pain. Possibility of this pain being related to the large bilateral adrenal myelolipomas was discussed. At this time the decision was made to continue to treat patient medically with oral prednisone therapy improving lab values. Patient continued to be followed with laboratory values at 5 and 6 months. Laboratory values at 5 months Androstenedione of 1020 (normal 40-150 ng/dL) and 17-hydroxyprogesterone of 7080 (normal <220 ng/dL) and at six months Androstenedione of 707 (normal 40-150 ng/dL) and 17-hydroxyprogesterone of 9390 (normal <220 ng/dL). With interval worsening of laboratory values despite medical management with prednisone, patient was reevaluated by surgical oncology for abdominal pain. Patient elected for surgical resection of the bilateral adrenal masses with the goal of symptom control.

**Fig. 5:**
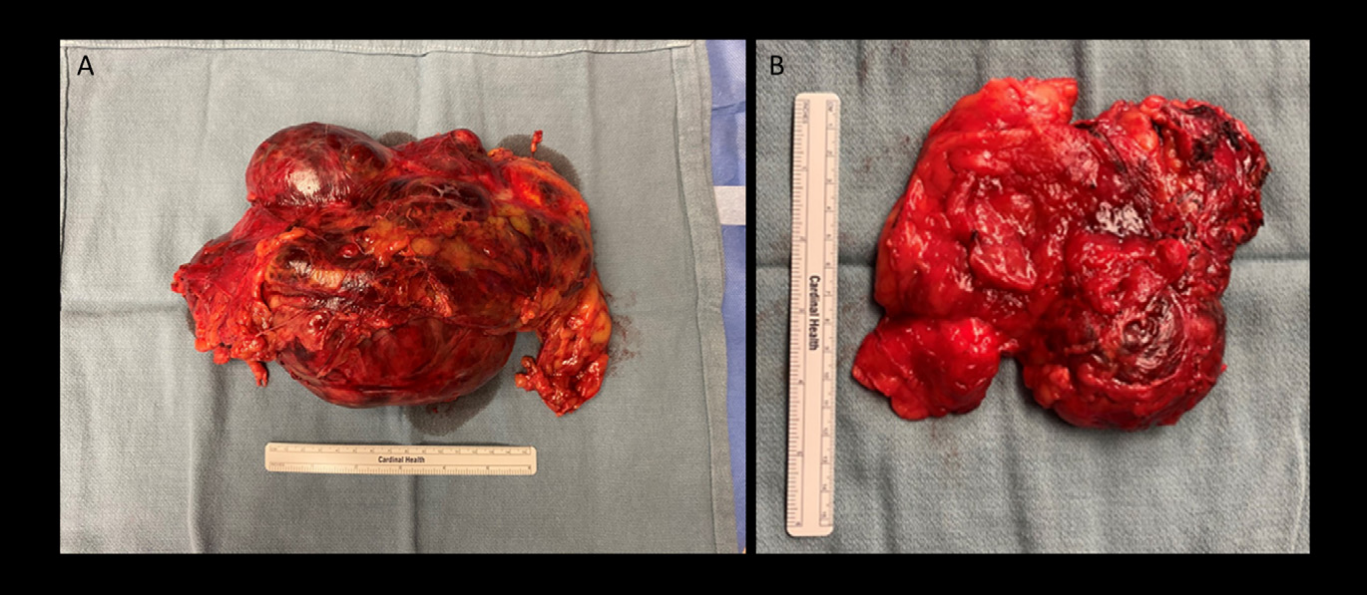
A: The right adrenal mass measures a 12.5 × 6.5 × 3.5 cm. The outer surface covered with adipose tissue and intact capsule. The outer surface reveals yellow-brown and hemorrhagic, fatty mass which is in contact with the gland. B: The left adrenal mass measures 23.5 × 19.5 × 6.5 cm, multinodular mass with intact capsule. The specimen reveals yellow-brown and hemorrhagic, fatty tissue which replaces the adrenal gland. Additional 3.5 × 3.4 × 1.9 cm, tan brown, firm, spherical mass which is in contact with the large mass.

**Fig. 6:**
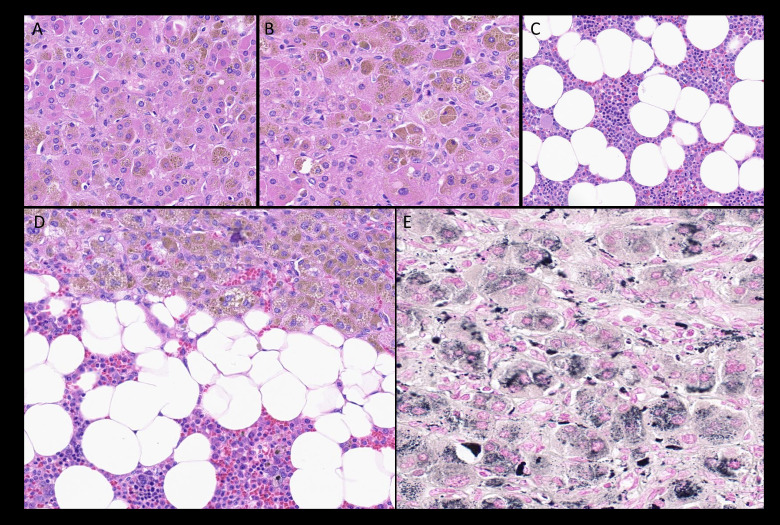
**A:** Lipid depleted cells with eosinophilic cytoplasm and abundant golden-brown lipofuscin of primary pigmented nodular adrenal disease. Hematoxylin and Eosin (H&E) (400x); **B:** Lipid depleted cells with eosinophilic cytoplasm and abundant golden-brown lipofuscin of primary pigmented nodular adrenal disease. H&E (400x); **C:** Myelolipoma is comprised of mature adipose tissue without atypia and mature bone marrow hematopoietic elements. H&E (400x); **D:** Myelolipoma with overlying rim of adrenal tissue. Myelolipomas are a known association with congenital adrenal hyperplasia (CAH) and primary pigmented nodular adrenal disease (PPNAD). H&E (200x); **E:** Fontana-Masson immunohistochemical stain (black chromogen) highlights the intracytoplasmic lipofuscin within lipid-depleted cells of PPNAD. (400x).

**Fig. 7:**
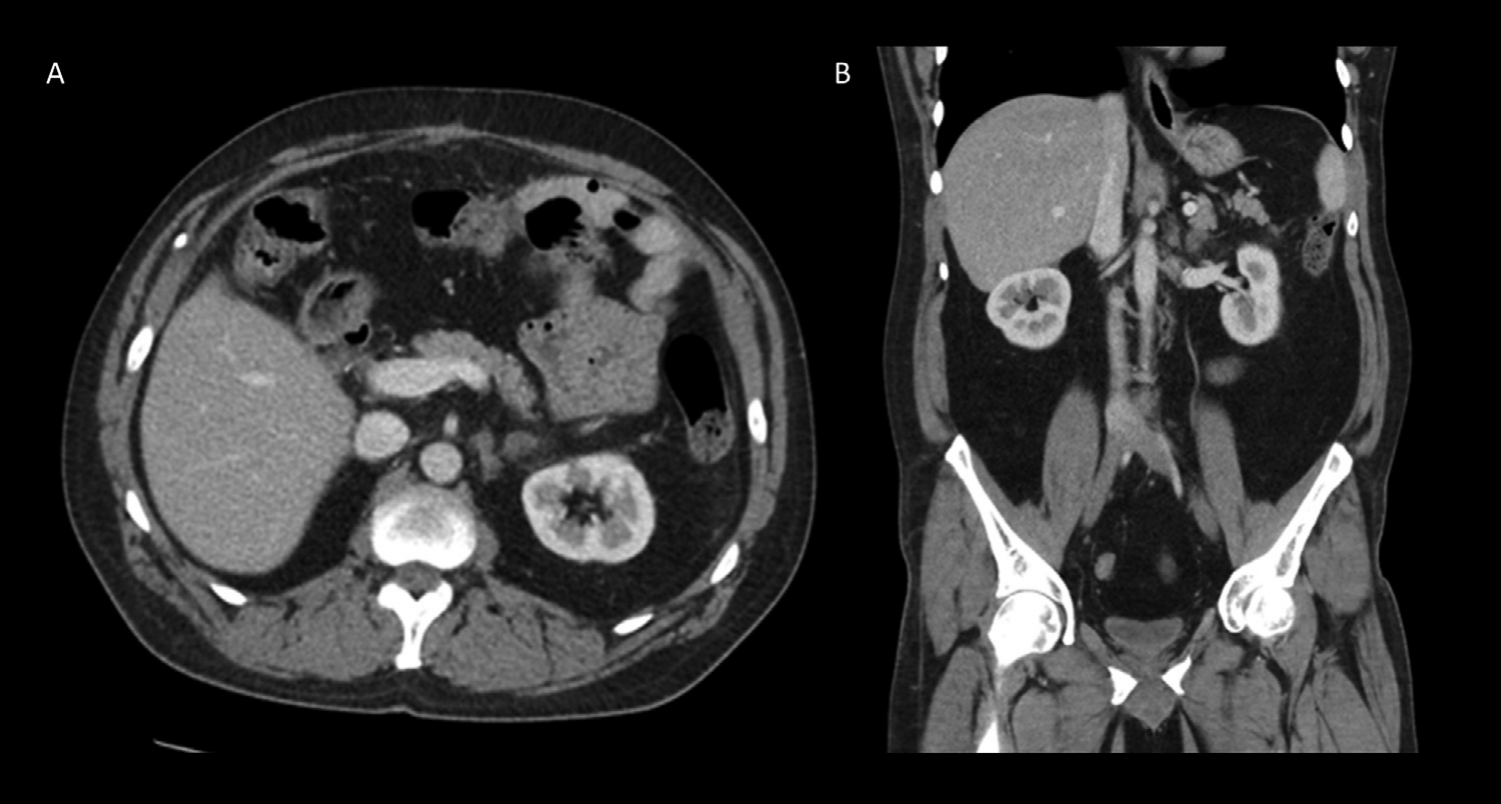
Postsurgical follow up CT, axial (Fig. A) and coronal (Fig. B) demonstrates nodularity at the left adrenal bed likely related to postsurgical change with recommendation for continued surveillance to monitor for recurrence.

**Fig. 8:**
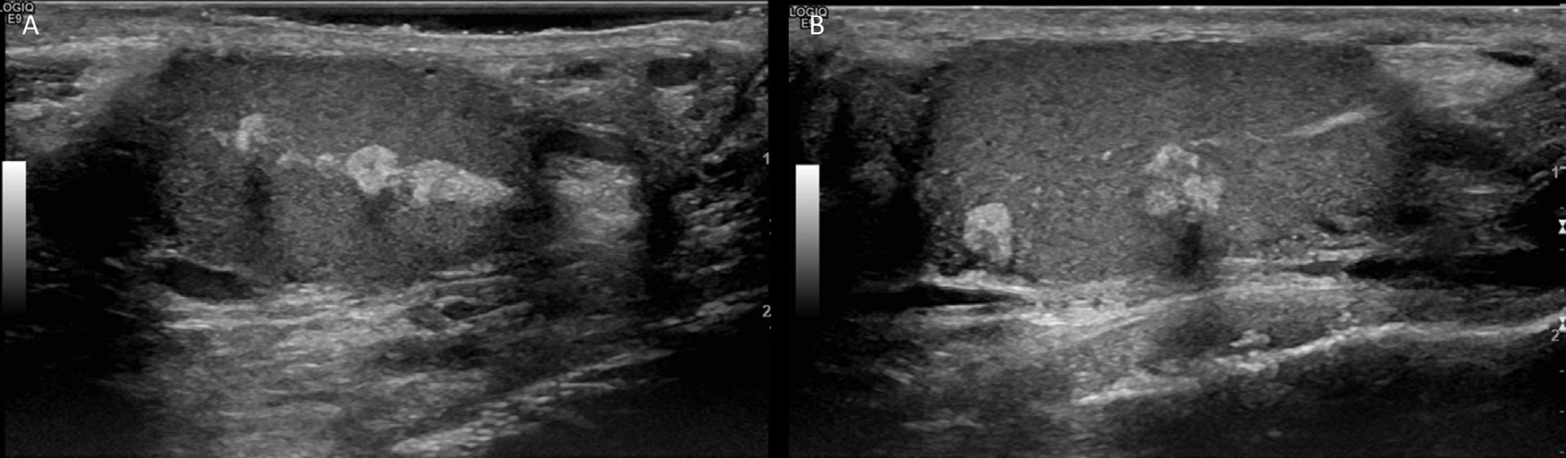
Follow up transverse testicular ultrasound in 3 mo, Right (A) and Left (B), displays unchanged size of the small mixed echogenicity masses in the bilateral testes.

Patient underwent bilateral adrenalectomy and pathology from the bilateral adrenal masses reported primary pigmented nodular adrenal disease with accompanying myelolipoma (Fig. 5, Fig. 6). Postoperative CT imaging at three months after surgery revealed nodularity at the left adrenal bed likely related to postsurgical change with recommendation for continued surveillance to exclude residual mass (Fig. 7). Repeat ultrasound evaluation of the scrotum revealed continued stability of the testicular lesions (Fig. 8) At time of publication, the patient is recovering well from adrenalectomy and continues to follow with endocrinology for exogenous steroid treatment.

## Discussion

Adrenal myelolipomas are benign tumors associated with congenital adrenal hyperplasia (CAH) especially in the setting of noncompliance with exogenous steroid treatment [1]. Though there is no definitive consensus of the pathogenesis of the giant adrenal myelolipomas, the findings may be explained by components of mesenchymal cells undergoing metaplastic change and/or overstimulation of adrenal cells via excess ACTH [1].

CT imaging findings display the constitutive histologic composition of adipocytes, appearing as well circumscribed hypodensity, and myeloid tissue, appearing as variegated increased density interspersed throughout the mass [6,7]. Complications include hemorrhage, rupture, necrosis, and mass effect on adjacent abdominal viscera [6,7]. Surgical resection is typically reserved for tumors greater than 6 cm in size due to increased risk of complication and symptomatology. Our case emphasizes the association of CAH with adrenal myelolipomas as well as emphasizes management considerations for masses measuring greater than 6 cm [7,8]. Testicular adrenal rest tumors (TARTs) are benign tumors associated with CAH [7]. Though there is not definitive consensus on pathogenesis, TARTs may be the result of embryological development in which aberrant adrenal cells descend to the testes and/or native testicular cells responding to excess in ACTH resulting in increased size [8]. Scrotal ultrasound is the imaging modality of choice for evaluation. Sonographic appearance is variable most often appearing as bilateral, multiple, heterogeneous, predominantly hypoechoic lesions located around the mediastinum of the testes [7,8]. Increased Doppler flow representing vascularity of the lesions is variable [7].

The main complication associated with TART is infertility as a result of testicular structural and blood flow impairment secondary to tumor burden [9]. Exogenous steroid treatment may provide mass stability or reduce TART's mass burden and reverse infertility [13]. The main differential diagnosis to consider for these lesions are Leydig cell tumors which be malignant and are difficult to distinguish from TART's histologically. Leydig cell tumors are typically unilateral and do not respond to steroids, differentiating them from TARTs. In the setting of testicular lesions with morphological features consistent with TART in a patient with known CAH these lesions are typically monitored with serial sonography. Understanding the association of TART to CAH and TART's morphological features can avoid unneeded patient anxiety, biopsy, and orchiectomy [9]. Our case emphasizes the association of TART to CAH, as well as reinforces the management of watchful waiting and avoiding unnecessary biopsy in management.

Primary pigmented nodular adrenocortical disease is an ACTH-independent cortisol producing lesion. PPNAD is the most common form of ACTH-independent micronodular hyperplasia or dysplasia that leads to increased cortisol production [1,2]. The hypercortisolism that results from PPNAD is due to autonomous secretion from nodules arising in the adrenal zona reticularis. Increased cortisol levels may lead to Cushing's syndrome, though manifestation is often subclinical. PPNAD is a rare cause of Cushing's syndrome and cases of PPNAD have been reported without Cushing's syndrome [1,2]. On gross appearance, the adrenal glands may be normal in size or enlarged and will contain multiple brown-black pigmented micronodules bilaterally, giving rise to the characteristic name of PPNAD. Nodules are unencapsulated with clear borders of demarcation due to adjacent atrophic cortex. Microscopically, cells are large and globular with clear or eosinophilic cytoplasm and may contain lipofuscin, which creates a granular brown pigment. Cells will stain highly positive for synaptophysin are consistent with neuroendocrine origin [10].

Cases of PPNAD may arise sporadically but is more commonly familial. The familial variant may arise as isolated PPNAD or be associated with Carney complex (CNC), with PPNAD being one of the major diagnostic criteria [2]. Diagnosis of PPNAD may be difficult in a patient with sporadic disease, no family history, lack of other clinical manifestations associated with CNC, and those with intermittent hypercortisolism. Suspected disease is evaluated in a similar manner to that of Cushing's syndrome by evaluating the plasma ACTH level and increased cortisol levels through 24-hour urinary free cortisol excretion, dexamethasone suppression test, and evening salivary cortisol [3,11]. While CT evaluation of the adrenal glands in a patient suspected to have PPNAD may show bilateral enlargement, nodularity, or characteristically a “strings of beads” appearance due to surrounding atrophic cortex, most cases of PPNAD will normal CT appearance of the adrenal glands [1,3]. Bilateral adrenalectomy is the recommended course of treatment for clinically diagnosed PPNAD [1].

This case report highlights the unique pathological findings in a patient with PPNAD who did not present with typical symptoms of Cushing syndrome and does not show any findings on imaging other than enlarged gland size most consistent with adrenal myelolipoma.

As briefly mentioned above, Carney complex, which occurs in association with PPNAD, is a multiple endocrine and nonendocrine neoplastic syndrome with associated skin findings. Cases of CNC can arise due to de novo germline mutations, but CNC also has an autosomal dominant inheritance pattern with high penetrance [5,11,12]. Two genetic loci have been identified that are responsible for the majority of familial cases and are located on chromosome 17q22-24 (causing inactivating mutations of the gene *PRKAR1A*) and chromosome 2p16 (*PRKAR1A*-negative patients) [4].

The endocrine neoplasms associated with CNC include primary pigmented nodular adrenocortical disease (occurring in 25%-60% of patients with CNC), asymptomatic growth hormone hypersecretion (up to 75%), large cell calcifying Sertoli cell tumor, and other pituitary, thyroid and ovarian tumors [1]. The non-endocrine neoplasms include cardiac myxomas, psammomatous melanotic schwannomas, and benign breast tumors –including myxomas, myxoid fibroadenomas, and ductal adenomas [1,5]. Skin findings seen in CNC include lentigines, which is found in 70%-80% of patients and is the one the first identifiable signs of CNC, especially if occurring on the vermilion borders of the lips, face, genitals, mucosa, and on the lacrimal caruncle/conjunctiva, which is an uncommon location for the general population [1,12]. Other common skin findings are blue nevi, café-au-lait macules, nevus spilus, spitz nevus, and cutaneous myxomas [1].

Diagnosis is made after clinical suspicion in patients presenting with the above cutaneous findings when 2 or more out of the major clinical manifestations of the disease are found through testing. If a first-degree relative is positive for CNC or there is an inactivating mutation of PRKAR1A gene, only one clinical manifestation is needed to make the diagnosis [12]. Treatment for CNC involves ongoing surveillance especially with cardiology follow up, as the greatest risk for mortality is cardiac disease secondary to cardiac myxomas which present with surgical complications and high reoccurrence rates [1,12].

While this case demonstrates a patient that presented with endocrine tumors, including that of PPNAD, at time of submission the patient was not evaluated further for Carney complex due to the lack of other clinical manifestations. Our case report emphasizes the important association between that of PPNAD and Carney complex, showing that PPNAD can arise sporadically or as an isolated case without the other manifestations of CNC.

## Patient consent

Informed consent was obtained from the patient.
